# Circadian Clock: A Regulator of Immunity in Autoimmune Diseases

**DOI:** 10.1002/iid3.70246

**Published:** 2025-09-05

**Authors:** Ye‐Jun Wu, Shu‐Ying Zhang, Hong‐Yu Chen, Xin‐Ran He, Guang‐Rui Yang

**Affiliations:** ^1^ School of Clinical Medicine Shanghai University of Medicine and Health Sciences Shanghai China

**Keywords:** autoimmune diseases, circadian clock, immune system, rheumatoid arthritis, type 1 diabetes

## Abstract

**Aim:**

Autoimmune diseases, characterized by the immune system mistakenly attacking the body's own tissues, are a growing global concern, with increasing prevalence. The circadian clock is a fundamental regulator of physiological processes, critically modulating immune functions. This review explores the intricate connections between circadian rhythms and immune responses in autoimmune pathogenesis and how disruptions exacerbate disease.

**Methods:**

This synthesis examines recent research on circadian regulation of immune functions (leukocyte trafficking, cytokine secreion, phagocytosis) and autoimmune progression. Key evidence includes roles of core clock proteins such as brain and muscle ARNT‐Like 1 (BMAL1), circadian locomotor output cycles kaput (CLOCK), and REV‐ERBα, along with circadian‐regulated immune cells, and impacts of environmental/lifestyle‐induced circadian disruption.

**Results:**

Ciradian rhythms significantly influence autoimmune disease progression and symptom patterns (e.g., morning joint stiffness in rheumatoid arthritis). Core clock proteins and rhythmic immune cells are critical for homeostasis. Circadian disruptions exacerbate immune dysfunction, promoting chronic inflammation and autoimmunity.

**Conclusions:**

The circadian clock is a fundamental regulator of immune function and autoimmune pathogenesis. Disruption worsens disease progression. Understanding these mechanisms opens new avenues for therapeutic interventions, including chronotherapy and targeting clock genes, with the potential to improve treatment outcomes in autoimmune diseases.

## Graphical abstract

This review explores the intricate connections between circadian clocks and immune responses in autoimmune diseases. We aim to open new avenues for therapeutic interventions, including chronotherapy and targeting clock genes, with the potential to improve treatment outcomes in autoimmune diseases.

## 1 Introduction

Autoimmune diseases are a group of inflammatory conditions where the immune system erroneously attacks the body's own tissues, involving autoreactive T cells or autoantibodies [1, 2]. Globally, the incidence and prevalence of these diseases have been rising annually by 19.1% and 12.5%, respectively. Common autoimmune diseases include Type 1 diabetes (T1D), systemic lupus erythematosus (SLE), rheumatoid arthritis (RA), and multiple sclerosis (MS) [3, 4]. Despite significant research, the precise mechanisms underlying the onset and progression of these diseases remain unclear, posing ongoing challenges for effective treatment.

The circadian clock, a molecular system governing biological rhythms over a 24‐h cycle, regulates various physiological processes, including immunity. For instance, the clock protein Rev‐erbα regulates lipid metabolism genes relevant to T helper 17 (Th17) cell development in the immune system [5]. Other clock components, such as nuclear factor interleukin 3 (NFIL3), circadian locomotor output cycles kaput (CLOCK), and retinoic acid‐related orphan receptor (ROR) α, are implicated in maintaining Th17 cell frequencies [5, 6], while brain and muscle ARNT‐Like 1 (BMAL1) is essential for B cell development [7], and Nfil3 is required for innate lymphoid cell (ILC) functionality [8, 9, 10]. Furthermore, the circadian clock modulates immune responses like leukocyte trafficking [11, 12], cytokine secretion [13, 14, 15], and macrophage phagocytosis [15, 16, 17], all of which exhibit circadian patterns.

Moreover, alterations in circadian oscillator levels or a loss of rhythmic immune responses can contribute to disease development [18, 19, 20], and given autoimmune diseases display circadian patterns in their symptoms, with these manifestations linked to underlying physiological or pathological rhythmic changes. For example, the morning stiffness and pain experienced by patients with RA have been associated with elevated nighttime levels of pro‐inflammatory factors in the synovium [21, 22]. Recently, more and more studies focused on the link between autoimmune diseases and circadian clocks, which would be helpful for the exploration on new treatment targets and strategies for autoimmune diseases. In this review, we explore the links between circadian clock dysregulation and autoimmune diseases, highlighting the potential of targeting circadian pathways for therapeutic interventions.

## 2 Overview of Biological Clocks

The circadian system is comprised of three parts: inputs, oscillators, and outputs and it operates at two levels, including systemic and cellular levels [23, 24].

At the systemic level, the master circadian pacemaker is located at the suprachiasmatic nucleus (SCN) of the hypothalamus [25]. Approximately 20,000 neurons among the SCN constitute a robust network, coordinating the neural and hormonal signals from other brain areas and peripheral tissues of the body and modulating rhythmic secretion of endocrine signals [25, 26]. In addition to the intrinsic modulation, external factors could also impact the circadian clock rhythmicity. The central clock responds to light input while other inputs including temperature, feeding, exercise, and humoral factors entrain peripheral clocks [27, 28, 29, 30]. The coordination of the SCN and peripheral clocks ensures the maintenance of intrinsic circadian timing in accordance with the external solar cycle and adaptation to the metabolic status of the peripheral tissues [31].

At the cellular level, the circadian clock system operates through two key transcriptional‐translational feedback loops [32]. The core loop includes four clock proteins, including Bmal1, Clock, periods (Per), and cryptochromes (Cry), in which Bmal1 and Clock transcription factor heterodimers promote the expression of *Per* and *Cry* by binding to the promotors of the genes. The encoded proteins then modulate the transcriptional activity and inhibit the expression of *Clock* and *Bmal1*. Since the two proteins (Per and Cry) are degraded via ubiquitin‐dependent pathways, their repression on the level of CLOCK/BMAL1 is terminated and this cycle has a ~ 24‐h periodicity [33, 34]. Another feedback loop is constructed through transcriptional activation by the RORs (RORα, β, γ) and transcriptional repression by Rev‐erbα/β [35, 36, 37], which promotes rhythmic changes in the transcription of *Bmal1* and drives a delay in the expression of *Cry1* that was essential to restore the circadian rhythmicity since the ROR‐REV feedback loop‐induced delay in the expression of *Cry1* is required for proper circadian timing when the Bmal1 abundance could not drive the core feedback loop [38, 39]. In addition, albumin D‐box binding protein (DBP) and the repressor NFIL3 also participate in the rhythmic feedback loop [40, 41].

Two components that are mainly involved in the circadian modulation include melatonin and cortisol. Melatonin is synthesized in the pineal gland. The absence of light contributes to the increased release of norepinephrine, and the activation of adrenergic receptors on the pinacocytes, which triggers intracellular responses and the activation of the enzymes N‐acetyltransferase and hydroxyindole‐O‐methyl transferase. The enzymes convert serotonin into melatonin [42]. In addition, the level of cortisol also exhibits a circadian rhythm that it rises in the early hours of the morning, peaks around 8 a.m., and has a nadir around noon, and then it starts to rise again [43].

### 2.1 The Circadian Clock and Immune System

The immune system is distributed throughout the body, including two branches: innate and adaptive immunity. Innate immunity is the frontline response against exogenous and endogenous danger signals in a nonspecific manner. In this process, classical innate immune cells, such as neutrophils, macrophages, dendritic cells, and innate lymphoid cells, are activated and some pro‐inflammatory factors are released [44]. Other than innate immunity, there is also infiltration of adaptive immune cells (T cells and B cells) in tissue inflammation. T cells receive signals from antigens and then differentiate into effector T cells, which produce pro‐inflammatory or anti‐inflammatory cytokines while B cells differentiate into plasma cells and produce specific antibodies upon stimulation [45].

**Figure 1:**
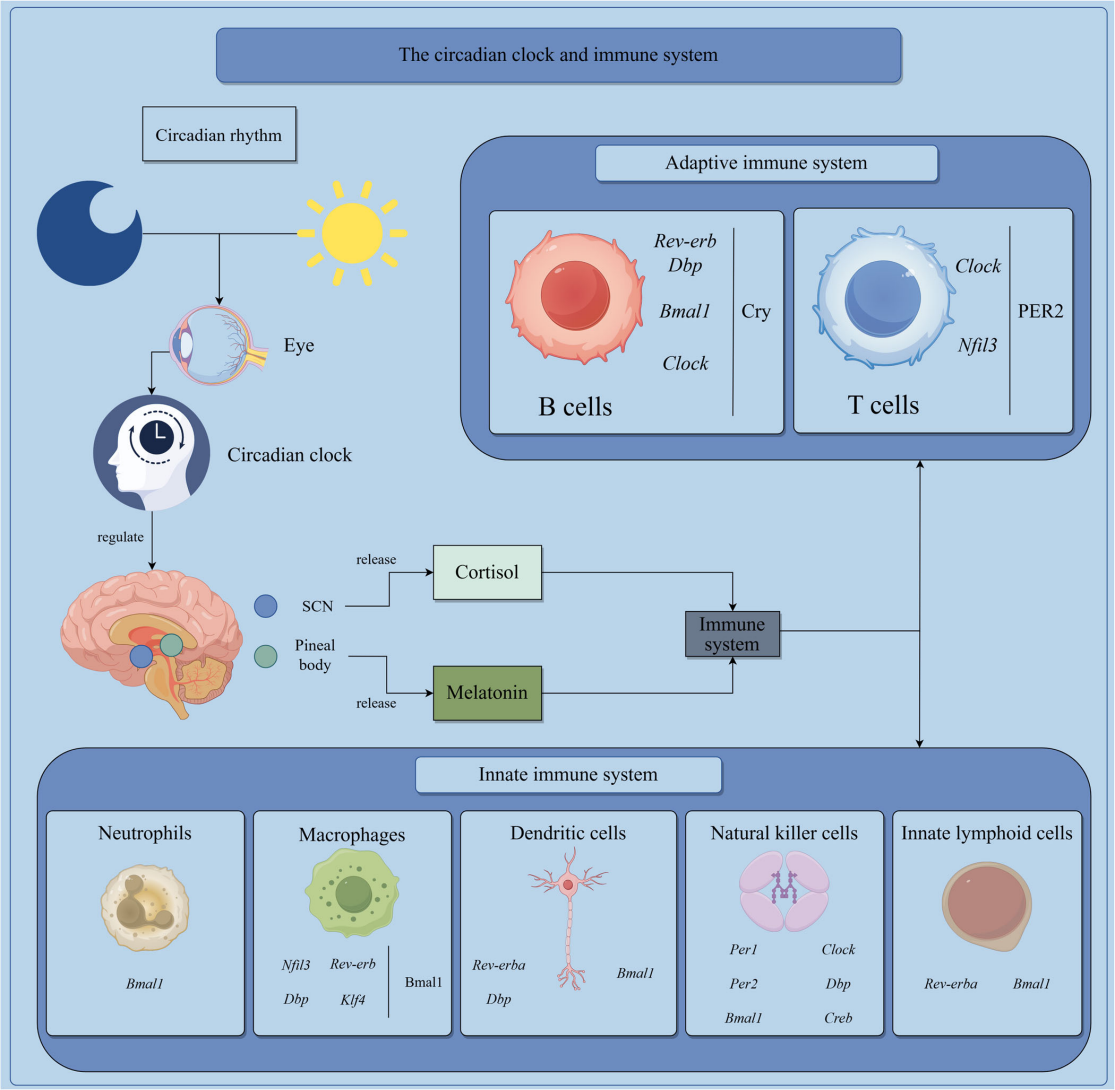
The circadian regulation on the immune system. By Figdraw (www.figdraw.com).

Melatonin and cortisol participate in the modulation of the circadian clock. Since melatonin and cortisol both have immunomodulatory effects [43, 46, 47], the immune activities exhibit circadian rhythmicity. Virtually all levels of immune function have circadian rhythms, including leukocyte development, leukocyte trafficking, and specific cell effects. We list the findings about circadian rhythms in immune activities by cell type (Table 1) and the regulation of circadian clock on immune systems (Figure 1).

**Table 1:** Circadian rhythms in immune activities by cell type.

Cell type	Organism of origin for the study	Finding about circadian rhythms	Pathogenesis of autoimmune diseases	Ref.
**Innate immunity**				
Neutrophils	Human	Expression of NADPH oxidase, superoxide production, and phagocytosis of opsonized bacteria all show daily oscillations.	Not reported	[48]
	Mouse	Bmal1 regulates expression of CXCL2 to induce CXCR2‐dependent diurnal changes in the transcriptional and migratory properties of circulating neutrophils.	Not reported	[49]
	Mouse	CXCL12/CXCR4 axis played an important role in the release and clearance of neutrophils in bone marrow.	Not reported	[50]
	Mouse	The aging of neutrophil was controlled by the clock gene *Arntl*, was entrained by light, and was dependent on antagonistic CXCR2 and CXCR4 signaling.	Not reported	[49]
	Mouse	Loss of *Bmal1* in neuptrophils ablated the rhythmicity of migration.	Not reported	[51]
	Mouse	Neutrophils regulate the homeostatic trafficking of hematopoietic precursors and allow synchronization of immune and hematopoietic rhythms.	DSS‐induced colitis	[52, 53]
Macrophages	Mouse	The expression of *Nfil3* and *Dbp* determine the production of cytokine IL‐12p40 from macrophages in response to LPS.	Not reported	[54]
	Mouse	Microglial *Rer‐erbα* deletion enhances inflammatory signaling, disrupts lipid metabolism, and causes lipid droplet accumulation specifically in microglia.	Not reported	[18]
	Mouse	Rev‐erb modulates the production of pro‐inflammatory cytokines including IL‐6 and regulates colitis via NF‐κB/Nlrp3 axis.	RA	[55, 56]
	Mouse	Inhibition of NOX2 enabled the microglia to retain a functional circadian clock while reducing levels of ROS and inflammatory cytokines.	Not reported	[57]
	Mouse	Deletion of the clock gene *Bmal1* leads to increased ROS and HIF‐1α levels, decreased activity of NRF2, increased production of pro‐inflammatory cytokine, and pyroptosis of macrophages.	Not reported	[13, 58, 59, 60]
	Mouse	Loss of rhythmic gene *Klf4* expression in aged macrophages is associated with disruption of circadian innate immune homeostasis.	Not reported	[61]
	Mouse	Phagocytosis has a circadian rhythm, is associated with the rhythms of mitochondrial dynamics, and is influenced by Bmal1.	Not reported	[61, 62]
ILCs	Mouse	*Bmal1‐*deficient ILC3s exhibit impaired expression of *Nr1d1* and *Per3*, hyperactivation of RORγt‐dependent target genes, and elevated proapoptotic pathways.	Not reported	[63]
	Mouse	ILC3‐autonomous ablation of the circadian regulator *Arntl* led to disrupted gut ILC3 homeostasis, impaired epithelial reactivity, a deregulated microbiome, increased susceptibility to bowel infection, and disrupted lipid metabolism.	Not reported	[64]
	Mouse	Rev‐erbα has circadian‐independent impacts on ILC3 development and function via regulating RORγt.	Not reported	[65]
	Mouse	Activation of the stress‐hormone‐sensing glucocorticoid receptor upregulates CXCR4 on ILCPs for their retention in the bone marrow, while the IL‐18 and RORα signals upregulate S1PR1 on ILC progenitors for their mobilization to the periphery.	Not reported	[14]
NK cells	Rat	Circadian changes in the expression of clock genes (*Per1*, *Per2*, *Bmal1*, *Clock*), *Dbp*, *Creb*, cytolytic factors and cytokines are observed in NK cells.	Not reported	[66]
	Mouse	*Per1* gene modulates immune pathways via NK cellular clocks.	Not reported	[67]
	Rat	Altered expression of clock genes, *Per2* and *Bmal1*, and cytolytic factors, perforin and granzyme B, as well as the cytokine, IFN‐γ was correlated with suppressed circadian expression of NK cytolytic activity.	Not reported	[68]
	Mouse	Chronic circadian disruption in NK cells (suppression of the expression of *Per1* and *Per2*) attenuated NK cell cytolytic activity by decreasing the expression of CD122.	Not reported	[69]
Mast cells	Human and mouse	Phosphorylation of ERK1/2, and expression of clock genes (*Per1*, *Per2*, *Cry1*, *Bmal1*, and *Clock*) show circadian rhythms in mast cells.	Not reported	[70, 71]
	Mouse	CLOCK temporally gates mast cell responses to IL‐33 via regulation of ST2 expression.	Not reported	[72]
	Mouse	The *Clock* mutation in mast cells resulted in the absence of temporal variations in IgE‐mediated degranulation in mast cells both in vivo and in vitro associated with the loss of temporal regulation of FcεRI expression and signaling.	Autoimmune‐like disease	[73]
DCs	Mouse	Circadian clocks control the infiltration of DCs into skin lymphatics.	Not reported	[11]
	Mouse	The expression of *Rev‐erbα* and *Dbp* experience robust oscillations in DCs.	Not reported	[74]
	Mouse	Circadian machinery in DCs contributes to the Th1/Th2 balance, and IL‐12 response to worm antigen by circadian‐synchronized DCs are dependent on Bmal1.	Not reported	[75]
	Mouse	Circadian clocks drive rhythmic antitumor immune responses mediated by migratory DCs.	Not reported	[76, 77]
**Adaptive immunity**				
B cells	Mouse	Oscillations of mRNA levels in the clock‐controlled transcription factors *Rev‐erbα* and *Dbp* are observed in mouse B cells.	Not reported	[74]
	Mouse	Bmal1 regulates the development of B cells.	Not reported	[7]
	Mouse	B cell development and the BCR‐signaling pathway are regulated by circadian clock Cry proteins.	Ulcerative colitis	[78]
	Mouse	Immunization during the period of lymphocyte accumulation in LNs enhanced antibody responses.	Not reported	[79]
	Human	CLOCK regulates IL‐10 expression in B cells in people with day‐night shift rotation.	Not reported	[80]
T cells	Mouse	Clock promotes T‐cell proliferation by modulating the timing of interaction between T‐cell and antigen‐presenting cells.	Not reported	[81]
	Mouse	Nfil3 suppresses Th17 cell development by directly binding and repressing the *Rorγt* promoter.	Not reported	[5]
	Mouse	The circadian clock of CD8 T cells modulates the response to vaccination by shaping the transcriptional program and making them more prone to efficient activation and proliferation according to the time of day.	Not reported	[82]
	Mouse	Genetic disruption of T cell clocks abolishes circadian regulation of lymphocyte migration.	Not reported	[12]
	Human	PER2 downregulated a disintegrin and metalloproteinase 12 (*ADAM12*) expression by reducing its binding activity, thereby suppressing IFN‐γ production in CD4 + T cells of ulcerative colitis.	Ulcerative colitis	[83]

Abbreviations: ADAM12, Disintegrin and metalloproteinase 12; BCR, B cell receptor; CXCL, C‐X‐C motif chemokine; CXCR, C‐X‐C motif receptor; DCs, dendritic cells; ERK1/2, extracellular signal‐regulated kinases 1 and 2; IFN, interferon; IL, interleukin; IROR, retinoic acid‐related orphan receptor; LC, innate lymphoid cell; LN, lymph node; LPS, lipopolysaccharide; NADPH, nicotinamide adenine dinucleotide phosphate; NOX2, NADPH oxidase isoform 2; NRF2, NF‐E2‐related factor‐2; ROS, reactive oxygen species; S1PR1, sphingosine 1‐phosphate receptor 1; NK, natural killer; Th, T helper.

The rhythmic characteristic of the immune circuit is the result of the coordinated action of neurons of the SCN, structural cells in tissues, and immune cells [84]. Circadian misalignment in various stages of rhythmic immune regulation might lead to immune disorders, and disease progression and exacerbation. A study focused on response patterns of immune rhythms in night shifters and pointed out that the altered pattern expressions of immune cells might increase susceptibility to infections and decrease the efficiency of vaccination in night workers [85]. In addition, it was indicated that in the case of chronic jet leg, the circadian clocks in immune cells were altered and thus the pace for cell growth was dysregulated, accompanied by a deficient or compromised immune system against tumor generation, leading to an increased risk of tumor development or progression [86, 87, 88]. It was also found that circadian disruption induced by light/dark cycle reversal promoted immune disorder, exacerbated renal fibrosis, and accelerated renal dysfunction, facilitating renal injury in chronic kidney disease [89]. There was also research highlighting the immunological effects of circadian dysregulation in chronic inflammation. Cheng et al. revealed that circadian disruption could help exacerbate allergenic reactions in the nasal mucosa and lung tissues by increasing Th2‐like immune response [90]. Moreover, intestinal inflammation was also reported to be associated with circadian clock dysfunction. It was found that light/dark phase shifts contributed to abnormal intestinal barrier function, leading to low‐grade intestinal inflammation [91]. Another research found that mice with colitis that had a lost circadian rhythm exhibited no daily rhythm in the infiltration of immune cells and worsened colitis severity [92]. Swanson et al. also suggested that circadian dysregulation was related to increased intestinal permeability, elevated blood pro‐inflammatory factors, and elevated intestinal inflammation in inflammatory bowel diseases [93]. In addition to inflammatory bowel disease, the role of circadian dysregulation in immunity in a lot of other autoimmune diseases has raised much interest, and we will discuss these in the following text.

Another important impact of circadian rhythm dysregulation on immunity lies in vaccine administration. The antituberculosis vaccine bacillus Calmette‐Guerin (BCG), influenza, and hepatitis A vaccine all showed a higher antibody response in the morning compared with immunization in the afternoon [94, 95, 96]. However, several studies showed different results that vaccination in the afternoon had higher responsiveness or the timing of vaccination did not affect the immune responsiveness [97, 98, 99]. Liu et al. indicated that age might influence the effects of the timing of vaccination and vaccination in the morning might enhance the immune responsiveness in adults over 65 years old [100]. Although there is currently no consensus on the best timing of vaccination, some studies have demonstrated the biological rhythm regulation involved in vaccine immunization. It was indicated that the circadian clock modulated the expression of Toll‐like receptor (TLR) 9. Therefore, using TLR‐9 as an adjuvant, immunization at the time when TLR‐9 had the highest activity might present an improved immune response [101]. Antigen processing also had circadian rhythms, and it was reported that oscillations in mitochondrial morphology and the associated metabolic output influence the antigen presentation process [102]. Ince et al. found a time‐of‐day‐dependent manner of the dendritic cell migration from the skin to the draining lymph node, which ensured the interaction between dendritic cells and T cells, and rhythmic expression of tumor necrosis factor and intercellular cell adhesion molecule‐1 (ICAM‐1), contributing to lymphocyte infiltration and lymph node expansion. It was suggested that lymph node cellularity governed the immune activities to vaccinations [103]. Nobis et al. used a vaccination model of antigen‐presenting cells loaded with an antigen to focus on T cell‐intrinsic functions and revealed the role of the CD8 T cell clock in the T cell response to vaccination [82]. Since there has been no definitive conclusion on the best timing of vaccination in clinical practice, further investigation on the mechanism and the layer of clinical setting is needed.

### 2.2 The Circadian Clock and Autoimmune Diseases

Autoimmune diseases are characterized as immune responses to self‐antigens, which contribute to damage to self‐organs or tissues. The etiologies of autoimmune diseases are complicated, and the widely accepted point is that the onset of autoimmune diseases is the result of the combined action of genetic predispositions, infection, and environment [104]. Previous studies have revealed higher incidences of rheumatoid arthritis (RA) [105], type 1 diabetes (T1D) [106], multiple sclerosis (MS) [107] in those who have a disturbance of circadian genes. Due to the circadian modulation in the immune system, circadian clocks have a role in the pathogenesis of autoimmune diseases. Considering the underlying links between autoimmune diseases and circadian clocks, we summarize the association between the circadian clocks and the immune changes in autoimmune diseases, prospecting for a better understanding of the pathogenesis of autoimmune diseases and potential synchronization therapy.

### 2.3 Rheumatoid Arthritis

RA is a chronic autoimmune disease, impairing many organs and tissues, and affecting polyarthritis (including large, medium, and small) systematically. The main characteristic of RA is synovitis and the inflammation in the synovial tissues contributes to the irreversible destruction of the joints [108]. Patients with RA exhibit diurnal variations in their symptoms, and the most representative manifestation is morning stiffness, which is recognized to be associated with increased pro‐inflammatory factors such as interleukin (IL)‐6 in the morning [109, 110].

Studies have also revealed several circadian genes involved in the modulation of rhythmic variations in RA. It was demonstrated that the Cry proteins had an inhibitory effect on the modulation of pro‐inflammatory tumor necrosis factor‐α (TNF‐α), as the deletion of *Cry1* and *Cry2* contributed to an increased production of TNF‐α [111]. Moreover, in *Cry1* ^*(‐/‐)*^ *Cry2* ^*(‐/‐)*^ mouse models, exacerbation of joint swelling and elevated inflammatory mediators such as TNF‐α and IL‐6 were observed [111]. In mouse models of anti‐type II collagen antibody‐induced arthritis, it was observed that the use of melatonin contributed to increased infiltration of inflammatory cells, exacerbation of synovial hyperplasia, and worsened articular cartilage destruction while markedly decreased expression of *Cry1*, suggesting that protein Cry1 might be involved in the melatonin‐mediated aggravation of joint inflammation and destruction [55]. Du et al. found that NFIL3 also contributed to RA pathogenesis as they observed elevated expression of NFIL3 in the peripheral blood and synovial tissues of RA patients, and an association between an increased NFIL3 level and abnormal inflammatory responses [112]. In turn, the inflammatory pathways could also directly affect the expression of clock genes. It was reported that TNF‐α induced the expression of *Bmal1* through calcium‐dependent pathways in synovial cells of RA [113]. Cavadini et al. found that TNF‐α and IL‐1β had inhibitory effects on the expression of *Per1/2/3*, *Dbp*, and *Tef* while promoted slightly the expression of *Clock* and *Rev‐erbα* [114]. Moreover, in vitro studies using RA‐FLS cells, it was observed that TNF‐α could inhibit the expression of *Per2* and promote the expression of *Clock*, *Bmal1*, and *Cry1* [115].

Systematic changes in circadian energy metabolism were also observed in RA. In chronic inflammatory diseases, immune activities require large energy consumption, which is crucial for energy regulation. In healthy individuals, energy allocation to immune activities is time‐dependent, as many aspects of immune activities (including antigen presentation, clonal expansion, and antibody production) are inhibited in the morning due to the immunosuppressive effects of cortisol and noradrenaline, and they are promoted in the midnight following low activities of the hypothalamic‐pituitary‐adrenal axis and the sympathetic nervous system [116, 117]. In parallel with waking up and increased levels of cortisol and noradrenaline in the morning, circulating cytokines are increased and lipolysis, β‐oxidation of fatty acids, glycogenolysis, and gluconeogenesis are stimulated [118]. However, in patients with RA, rhythmic metabolic rewiring was observed as β‐oxidation and lipid handling were impaired while sphingolipid and ceramide were accumulated [119]. It was demonstrated that the arthritis‐related production of ceramides was most pronounced and it was at the time of peak inflammation [119]. Since the circadian symptoms of RA arise during the night when energy consumption is allocated for increased immune activities and ceramide accumulation accompanied by peak inflammation, energy redistribution might be a promising treatment target for patients with RA and it deserves further investigation.

Modified prednisone releases were indicated in patients with active RA: plasma cortisol levels started to rise at 23:00–02:00, earlier than healthy controls, and had a higher peak [120]. This earlier rise of cortisol was preceded by a rise in IL‐6, which was tightly associated with the pathogenesis of RA including the clinical manifestations such as joint swelling and stiffness [43, 120]. IL‐6 also played a role in stimulating the cortisol secretion [43]. Based on this biorhythm in RA, a study compared the treatment effects of using prednisone at 2:00 a.m. with that at 7:30 a.m. and found improved morning stiffness, pain, and disease activity when using prednisone at 2:00 am [121]. Another study used a novel modified‐release prednisone tablet at 10:00 p.m. which released prednisone at 2:00 a.m. and found significant improvement in clinical manifestations [122]. Moreover, Buttgereit et al. assessed the efficacy and safety of the modified‐release prednisone compared with standard prednisone and found that patients receiving the modified‐release prednisone achieved a mean reduction of 44 min in the duration of morning stiffness, and the absolute difference between the two groups was 29.2 min, indicating a better efficacy of the modified‐release prednisone (*p* = 0.072) [123]. Since morning stiffness is one of the early symptoms recommended for diagnosis of RA and is associated with abnormal immune rhythms, it is of great importance to carry out multi‐center large‐scale studies to investigate the efficacy and safety of treatment strategies that consider the timing of inflammatory activities.

### 2.4 Type 1 Diabetes

T1D derives from a progressive loss of pancreatic β‐cell function and requires daily insulin treatment. T1D is mediated by a cell‐mediated immune response, which is driven by aberrant T cells, causing the loss of self‐recognition of T cells and targeting the antigens of pancreatic β cells [124]. Previous studies have revealed that circadian oscillators play a role in the pathogenesis of T1D. Hofmann et al. investigated the rhythmic expression of clock genes and clock‐controlled genes in rat models with T1D including streptozotocin‐treated rats, spontaneous T1D LEW.1AR1‐iddm (lddm) rats and lddm rats used insulin for 10 days, and found that the clock genes all exhibited a rhythmic expression pattern while the expression of *Bmal1*, *Per2*, and *Clock* was altered in lddm and insulin‐treated lddm rats [125]. Furthermore, using insulin helped normalize the rhythm in the expression of *Dbp*, *Rev‐erbα*, and *E4bp4* [125]. Marcheva et al. also observed oscillations of the expression of *Clock* and *Bmal1* in pancreatic islets, and they found that conditional knockout of the pancreatic clock genes (including *Clock* and *Bmal1*) contributed to insulin deficiency, glucose tolerance impairment, and the onset of diabetes [126]. In addition, the aryl hydrocarbon receptor (AHR), a member of the family of the homologous genes of the transcriptional‐translational feedback loops, was demonstrated to be associated with the pathogenesis of T1D [127]. It was suggested that AHR had a role in the development of Treg and Th17 [128, 129]. Moreover, the use of AHR ligand 2,3,7,8‐Tecrachlorodibenzo‐p‐dioxin (TCDD) in Nonobese diabetic (NOD) mice could reduce the pancreatic islet insulitis with an expansion of Treg in the pancreatic lymph nodes, which indicated a suppression in the development of T1D [127]. Aryl hydrocarbon receptor nuclear translocator‐like 2 (ARNTL2), also known as Bmal2, was suggested to contribute to variation in the regulation of circadian rhythm [130, 131]. In NOD mice, *Arntl2* was indicated to be a candidate gene for T1D as the expression of *Arntl2* was downregulated in NOD mice compared with that in nondiabetic mice [132, 133]. It was also demonstrated that Arntl2 could bind to the promotor of the *Il‐21* gene to control the expression of *Il‐21* without interaction with other oscillators such as Bmal1 or Clock [134]. IL‐21 could control the proliferation of immune cells, and the gene *Il‐21* is located at the T1D locus *Idd3* [135, 136]. It was shown that IL‐21 was associated with the pathogenesis of T1D [137], and it was shown to have an increase in the number of IL‐21‐secreting effector T cells in patients with T1D [138]. However, there is no data about the circadian changes of ARNTL2 or IL‐21 in T1D. Another study revealed the circadian dysrhythmia of immune cells of T1D, as patients with T1D had significant phase shifts in the time of peak occurrence of B cells ( + 4.8 h), CD4 and CD8 T cells ( + 5 h), and their naive and effector memory subsets ( + 3.3 to +4.5 h), and regulatory T cells ( + 4.1 h) [139]. Further investigations are needed to find the diurnal changes in the interaction between circadian oscillators and immunity in T1D.

### 2.5 Multiple Sclerosis

MS, an immunological disease that is characterized by chronic inflammation and acute inflammatory lesions in the central nervous system (CNS), could lead to neural demyelination and neurodegeneration [140, 141]. It has been generally accepted that autoimmunity plays a major role in the pathogenesis of MS, and specific T cells and B cells are both involved in mediating the disease [142, 143]. MS includes three subtypes: relapsing‐remitting subtype of MS (RRMS) which is characterized by recurrent episodes of neurological dysfunction caused by acute inflammatory demyelination, primary progressive MS (PPMS), and secondary progressive MS (SPMS) that is defined as progressive neurodegeneration leading to neurological disability [144]. Previous studies supported a significant association between shift work and increased MS risk [145, 146]. Moreover, it was revealed that insufficient sleep and low sleep quality might increase the risk of developing MS [147, 148]. These findings suggested that MS might be associated with a dysregulation of circadian rhythms.

Several clock genes have been reported to be associated with MS. The polymorphism of genes *ARNTL, CLOCK*, and *PER3* were all reported to be associated with MS risk or MS exacerbation [149, 150]. Six genes (*CAMK2G*, *GRIN2A*, *GRIN2B*, *N1RD1*, *PER3*, *PPARGC1A*) encoded for circadian entrainment components were shown to be associated with MS [151]. Buenafe also found that the diurnal rhythm of *Per2* expression was altered in mice with EAE [152]. Moreover, Sutton et al. used a mouse model of experimental autoimmune encephalomyelitis (EAE), an animal model of MS, and found that Bmal1 regulated the accumulation and activation of immune cells in EAE, and the loss of myeloid Bmal1 exacerbated inflammation in the CNS through the expansion of IL‐1β secreting monocytes, which increased pathogenic IL‐17^+^/IFN‐γ^+^ T cells [107]. Although data on the association between circadian clock genes and immunity are limited, the modulation of Bmal1 on inflammation in CNS of EAE indicated that circadian dysfunction might result in immunity disruption and malfunction in MS. More studies are needed to investigate the link between circadian disruption and immune cell dysfunction in MS.

It was indicated that the level of melatonin was decreased in patients with MS [153]. Kern et al. observed that lower melatonin levels within the first hour after awakening were indicative of longer MS duration. They also found that melatonin levels correlated with neurological disability, fatigue, and health‐related quality of life [154]. The findings of Damasceno et al. also supported that the disruption of melatonin circadian rhythm production was associated with disability and fatigue [155]. Several studies also investigated the circadian rhythm of cortisol in MS. An increase in the cortisol awakening response and an increase in cortisol within approximately 30 min after awakening were found, and this change in the cortisol level was found to be associated with neurological disability worsening and fatigue [156, 157, 158]. However, the association between immunity and the changes in melatonin and cortisol levels in MS has not been studied yet.

MS symptoms also showed diurnal variations. It was observed that fatigue and pain increase over the day while muscle strength, objective, and subjective cognitive performance decrease over the day [159, 160]. The reason why MS symptoms have circadian rhythmicity has not been investigated yet. Davis et al. indicated that circadian temperature variations might be associated with the fluctuation of motor function [161]. More studies are needed to investigate the underlying circadian mechanisms.

### 2.6 Other Autoimmune Diseases

Systemic lupus erythematosus (SLE) is a chronic connective tissue disease, characterized by loss of immune tolerance to self‐antigens, and the formation of immune complexes [162]. Dan et al. performed a case‐control study and found that *PER2* gene single‐nucleotide polymorphisms were related to the SLE pathogenesis [163]. Moreover, IL‐17A was suggested to be involved in the development and progression of SLE, and it was found that the level of IL‐17A was increased in patients with SLE [164, 165]. The expression of IL‐17A required RORγt [166], a receptor activates transcription and participates in circadian rhythms [167]. However, there is no research concerning the role of clock genes, especially the ROR family in SLE. Existing data on the association between circadian modulation in immunity and the pathogenesis of SLE are also scarce and more research is warranted.

Helvaci et al. provided the first evidence for an association between autoimmune thyroid disease and clock gene *PER3*, indicating the possible relevance of *PER3* gene polymorphisms in the pathogenesis of autoimmune thyroid disease [168]. Ando et al. also established a link between psoriasis and the circadian clock, as they found that Clock could regulate psoriasis‐like skin inflammation in mice models via modulation of the expression of IL‐23R in γ/δ + T cells [169]. Lizuka et al. revealed the crucial role of RORγt in the development of Sjogren's syndrome [170]. However, data on circadian modulation in the pathogenesis of these diseases are limited.

Recent studies linked the association between circadian disruption and intestinal dysbiosis in the pathogenesis of inflammatory bowel disease (IBD). Genetic deficiencies in various circadian clock components (PER1/2, BMAL1, CLOCK, RORα, and REV‐ERBα) have been linked to an increased susceptibility of IBD mainly via influencing colonic permeability by regulating the integrity of the intestinal barrier [56, 83, 92, 171, 172, 173]. Cao et al. also revealed the role of the modulation of Cry proteins in B cell development and the BCR‐signaling pathway in ulcerative colitis [78]. In addition, mice with induced colitis and patients with IBD also exhibit disruptions in clock gene expression (including genes encoding BMAL1, CLOCK, PER1/2, CRY1/2, NPAS2, REV‐ERBα, RORα, etc.) [56, 174, 175]. Moreover, there are also studies investigating the association between circadian disruption and intestinal dysbiosis in the pathogenesis of extra‐autoimmune diseases, which mainly focused on RA. It was suggested that barrier permeability was affected by arthritis and increased barrier permeability during disease initiation is reported to be accompanied by increased frequencies of Th1 and Th17 cells in the small intestines, which have the potential to migrate to the joints [176]. Moreover, temporal remodeling not only in the microbial composition of the gut [*Alistipes* and *Odoribacter* (both *Bacteroidetes*) and *Lactobacillus* (a *Firmicute*)] but also metabolic outputs (indolelactate and methyl indole‐3‐acetate) was observed in collagen‐induced arthritis [177]. However, the mechanisms underlying crosstalk between the inflamed joint, the colon and its constituents are unknown and need further investigation. These findings might also indicate new directions for the study of the association of circadian clock and intestinal homeostasis in other extra‐intestinal autoimmune diseases since intestinal dysbiosis was reported to be related with many autoimmune diseases including MS, T1D, and SLE [178].

Immune thrombocytopenia (ITP) is an autoimmune bleeding disease characterized by decreased platelet counts and an increased bleeding risk [179]. The pathogenesis of ITP includes decreased platelet production by megakaryocytes in the bone marrow and increased platelet destruction [179]. A previous study demonstrated that the *Per2* gene was required for platelet formation [180]. Moreover, it was indicated that the total platelet numbers and plasma thrombopoietin (TPO) concentration were in circadian rhythms and entrained by the light‐dark cycle [181]. Tracey et al. observed that CLOCK regulated the diurnal expression of the TPO gene in human cell lines [182]. The above studies provided evidence that platelet production was in circadian modulation, and further studies are needed to investigate whether there are circadian changes in ITP, which might provide new treatment targets for ITP.

### 2.7 Circadian Modulation in Autoimmune Disease Treatment

Remarkable progress has been made in the understanding of the circadian clock in innate and adaptive immunity, and autoimmune diseases in the last few years. Therefore, this evidence might support the idea of modulating or maintaining circadian rhythms for treating diseases [183].

Many clock genes have been demonstrated to influence immunity and the pathogenesis of autoimmune diseases, among which *RORs* might be the most promising clock genes to act as therapeutic targets. Previous studies have demonstrated that RORs are responsible for regulating Th17 differentiation and regulating the IL‐17 pathway [184, 185, 186]. RORγt, a subtype of RORγ, is not only a key circadian rhythm regulator but also a crucial transcription factor of immune cells. Recently, RORγt has attracted much interest as a potential therapeutic target in autoimmune diseases. Digoxin, SR1001, SR1555, TMP778, and TMP920 were all proposed to display effects on RORγt and further regulated immunity [187, 188, 189, 190]. Moreover, another important circadian modulator, Rev‐erbα, is suggested to act as an antagonist of RORγt and regulate the differentiation of Th17 [191]. Therefore, REV‐ERBα might also serve as a potential treatment target for autoimmune diseases. There have been some studies to investigate the agonist of REV‐ERBα in autoimmune diseases, and the involved agents include GS2667, GSK5072, GSK2945, SR9009, and so on [192, 193]. However, these studies have all focused on the effects of the agents on the differentiation of Th17 cells and cytokine secretions. No research has focused on the circadian regulation of the immune system under the condition of autoimmune diseases by these agents, which deserves more research attention.

Factors reported to have an impact on the circadian system include light, food, exercise, and hormones (including melatonin and cortisol), et al., which have been investigated as treatment approaches to reset or bolster the circadian rhythms in many diseases [194, 195, 196, 197]. In autoimmune diseases, there have also been some reports. In a study using collagen‐induced arthritis mice models, chronotherapy with Baricitinib could target the secretion of cytokines and was demonstrated to be effective in the treatment of arthritis [198]. Dietary approaches, such as intermittent fasting, which means alternate day fasting involving a combination of no‐eating days with eating days, have also been investigated and were indicated to impact circadian rhythms and improve inflammatory parameters in MS [199]. However, there are also negative results. De Carvalho et al. summarized the efficacy of melatonin supplementation in rheumatological disease and found that melatonin supplementation was not effective in RA and SLE [200]. A series of clinical trials are currently being conducted to explore the effects of zeitgeber regulation on circadian rhythms and disease progression itself in autoimmune diseases. For example, a study focused on the effects of light therapy in progressive MS (NCT 06261528). Another study aimed to determine the effects of intermittent fasting and a Mediterranean diet in patients with MS (NCT 06546033). Furthermore, melatonin and cortisol are both vital hormones that are involved in circadian regulation. Considering the changes in the two hormones observed in autoimmune diseases, melatonin and corticosteroid treatment for autoimmune diseases are proposed. Indeed, corticosteroids are the first‐line or recognized effective treatment of many autoimmune diseases, including RA, and SLE. However, due to the changes in the circadian rhythmicity of corticosteroids in disease conditions [120], further studies on the best timing of treatment are needed. Melatonin supplementation is also explored in autoimmune diseases, such as MS, and is indicated to improve sleep in patients with MS [201]. However, there are still not many related studies available. Taken together, there have been limited data on the effects of zeitgeber modulation on immunity and autoimmune diseases, and more studies are needed to find new treatment targets.

## 3 Conclusion

Circadian clocks play a fundamental role in regulating immune function, and numerous circadian alterations have been observed, including changes in clock gene expression, immune cell function, cytokine secretion, hormone levels, and disease symptoms in autoimmune diseases. Targeting clock genes or modulating circadian rhythms could offer new therapeutic strategies to improve immune stability and manage autoimmune conditions. Currently, research on targeting circadian rhythm regulators for the treatment of autoimmune diseases mainly focused on RORγt and REV‐ERBα, which might have the greatest potential for clinical application. As the links between circadian biology and immunity become clearer, further research is urgently needed to explore the therapeutic benefits of circadian modulation in autoimmune diseases and uncover the underlying immunological mechanisms. Potential adverse reactions should also be considered due to the involvement of the circadian clock in regulating various physiological functions.

## Author contributions

**Ye‐Jun Wu:** conceptualization, formal analysis, project administration, resources, writing – original draft. **Shu‐Ying Zhang:** resources, supervision, writing – review and editing. **Hong‐Yu Chen:** resources, writing – review and editing. **Xin‐Ran He:** writing – review and editing. **Guang‐Rui Yang:** conceptualization, methodology, project administration, supervision, writing – review and editing.

## Conflicts of Interest

The authors declare no conflicts of interest.

## Data Availability

The authors have nothing to report.
